# Evaluation of serotonin and serotonin transporter levels among Obstructive Sleep Apnea patients

**DOI:** 10.1192/j.eurpsy.2023.604

**Published:** 2023-07-19

**Authors:** A. Gabryelska, D. Strzelecki, M. Sochal

**Affiliations:** ^1^Department of Sleep Medicine and Metabolic Disorders; ^2^Department of Affective and Psychotic Disorders, Medical Univesity of Lodz, Lodz, Poland

## Abstract

**Introduction:**

Obstructive sleep apnea (OSA) is characterized by recurrent pauses in breathing during sleep leading to sleep fragmentation and further excessive daytime sleepiness. Therefore, OSA patients are at high risk of suffering from complications from psychiatric disorders. Serotonin is a known neurotransmitter and together with serotonin transporter (SERT) is involved in the development of depression and insomnia.

**Objectives:**

The study aimed to evaluate serotonin and SERT levels among OSA and healthy individuals and their association with insomnia and depression symptoms.

**Methods:**

Forty individuals following polysomnography (PSG), based on the apnea-hypopnea index (AHI), were divided into 2 groups: the OSA group (AHI30; n=20) and the control group (AHI<5; n=20). Participants filled out questionnaires: Beck Depression Inventory (BDI) and Athens Insomnia Scale (AIS). Peripheral blood was collected in the morning after PSG. Protein concentrations were measured using ELISA. Further groups were divided into subgroups based on the standard cut-off points: without (AIS (-)) and with (AIS (+)) insomnia symptoms (AIS>5) and without (BDI (-)) and with (BDI (+)) depression symptoms BDI (BDI>19).

**Results:**

No differences between the OSA and control groups in serotonin (128.8 (73.4 – 209.0) vs. 132.7 (69.9 – 214.6) ng/ml, p=0.805 and SERT (55.8 (39.7 – 64.80) vs. 576.4 (424.2 – 658.3) pg/ml, p=0.564) levels were observed. In OSA group SERT level correlated with AHI (r=0.409, p=0.043), desaturation index (r=0.504, p=0.024) and mean oxygen desaturation during night (r=-0.522, p=0.018), while serotonin level was associated with BMI (r=0.550, p=0.012), but not PSG parameters. Serotonin level was higher in the AIS (+) group but only in healthy individuals. Further, serotonin levels decreased in the BDI (+) group, yet this finding was observed only in the control group.

**Conclusions:**

The results show that serotonin levels are associated with the presence of insomnia in depression, but quite interestingly only among healthy individuals. The association between oxygen desaturation and SERT levels suggests the involvement of hypoxia in the serotonin signaling pathway. Yet further studies on larger populations are needed to better understand the mechanisms responsible for the development of psychiatric complications in OSA patients.

**Disclosure of Interest:**

None Declared

